# Subconscious Visual Cues during Movement Execution Allow Correct Online Choice Reactions

**DOI:** 10.1371/journal.pone.0044496

**Published:** 2012-09-25

**Authors:** Christian Leukel, Jesper Lundbye-Jensen, Mark Schram Christensen, Albert Gollhofer, Jens Bo Nielsen, Wolfgang Taube

**Affiliations:** 1 Department of Sport Science, University of Freiburg, Freiburg, Germany; 2 Department of Medicine, Movement and Sport Science, University of Fribourg, Fribourg, Switzerland; 3 Department of Exercise and Sport Sciences, University of Copenhagen, Copenhagen, Denmark; 4 Department of Neuroscience and Pharmacology, University of Copenhagen, Copenhagen, Denmark; 5 Danish Research Centre for Magnetic Resonance, Copenhagen University Hospital Hvidovre, Copenhagen, Denmark; University of Chicago, United States of America

## Abstract

Part of the sensory information is processed by our central nervous system without conscious perception. Subconscious processing has been shown to be capable of triggering motor reactions. In the present study, we asked the question whether visual information, which is not consciously perceived, could influence decision-making in a choice reaction task. Ten healthy subjects (28±5 years) executed two different experimental protocols. In the *Motor reaction protocol*, a visual target cue was shown on a computer screen. Depending on the displayed cue, subjects had to either complete a reaching movement (go-condition) or had to abort the movement (stop-condition). The cue was presented with different display durations (20–160 ms). In the second *Verbalization protocol*, subjects verbalized what they experienced on the screen. Again, the cue was presented with different display durations. This second protocol tested for conscious perception of the visual cue. The results of this study show that subjects achieved significantly more correct responses in the *Motor reaction protocol* than in the *Verbalization protocol*. This difference was only observed at the very short display durations of the visual cue. Since correct responses in the *Verbalization protocol* required conscious perception of the visual information, our findings imply that the subjects performed correct motor responses to visual cues, which they were not conscious about. It is therefore concluded that humans may reach decisions based on subconscious visual information in a choice reaction task.

## Introduction

A question that fascinates both psychologists and scientists interested in motor control is whether our behaviour can be influenced by sensory information that is not consciously perceived. So-called ‘subliminal priming studies’, where a subliminal prime is presented before a supraliminal target cue, have shown that responses can be facilitated or inhibited by prime stimuli presented below the threshold for conscious perception [1], [2], [3]. In these studies, the visual stimulus is manipulated in order to affect the perceptibility and preclude conscious perception. One way to provide subliminal visual cues is to consecutively reduce the display duration of the visual stimulus. It is believed that, using sufficiently short display duration, the visual cue is prevented from entering conscious perception.

In priming studies, the second, consciously perceivable stimulus, has often been used to trigger a specific reaction (e.g. when seeing a light a subject has to reach for a button as quickly as possible) and the previous weaker stimulus may bias the reaction (e.g. the reaction to the button may become quicker or slower than without the first cue). This biasing or priming was intensively studied using a simple reaction time task [3]. Changes in the reaction time in these studies were interpreted as evidence that subconscious visual information affects the motor reaction and therefore plays a role for the execution of the motor response.

Further evidence that subconscious visual information may be used for motor control comes from studies in patients describing the so-called “blindsight behaviour”. One of the first reports describes the case of a female patient with damage of the primary visual cortex who was able to perform accurate reaching movements to visual stimuli without conscious visual perception [4]. Subsequently, additional studies have also been performed with healthy subjects to test their ability to react to visual cues, which they did not consciously perceive. This has been accomplished by temporarily “knocking out” the primary visual cortex by means of transcranial magnetic stimulation [5]. Applying this method, the experiment of Christensen et al. (2008) demonstrated that healthy subjects were able to adequately perform a reaching correction task without consciously experiencing the visual stimulus guiding the movement.

However, although it is generally accepted that not only visual but also auditory [6], somatosensory [7] and olfactory [8] sensory information can be processed at intensities below conscious perception, there is still an on-going debate about whether and how subliminal stimuli can guide decision making [9], [10]. With respect to visual information, Taylor and McCloskey [11] applied a choice reaction task in order to clarify this point. They positioned two pairs of light-emitting diodes (LEDs) left and right to a central visual fixation point. Subjects should perform a different movement in response to activation of the left LEDs than after flashing the right LEDs. A subconscious prime cue (consisting of weak LEDs) was presented 50 ms before a consciously visible cue (consisting of strong LEDs surrounding the weak LED). When the first cue (weak LEDs) was presented shortly before the conscious one (strong LEDs), subjects displayed a faster reaction time compared to the conscious stimulus alone (only strong LEDs). Therefore, the authors concluded that subconscious information may not only be used for initiating motor responses in simple tasks but also to choose between alternative motor actions.

The idea that the brain is able to utilize subconscious visual information to select between different motor responses is indeed appealing. However, an open question remains: Is a subconscious cue alone sufficient as a basis for choosing between two possible motor responses? In the study of Taylor and McCloskey [11], the presentation of the subconscious cue 50 ms before the strong stimulus indeed shortened the reaction time. However, the experiment could not clarify whether the subconscious cue indeed initiated the response or whether it only facilitated the reaction (in terms of a faster reaction time) that is actually initiated in response to the strong stimulus (mask). This is because the mask was always exclusively presented on the same side as the subconscious cue, not on both sides (i.e. it was not a “neutral” mask). Both stimuli, the subconscious (first) and conscious (second) one, therefore had the same meaning.

Consequently, the aim of the present study was to clarify whether a subconscious stimulus on its own is sufficient to trigger appropriate motor responses in a choice reaction task. In contrast to previous studies, we provided single subconscious cues with different information content than the masking stimulus. More specifically, only the information content of the subconscious cue indicated how subjects should react, and the masking stimulus had a neutral (non-informative) meaning. We displayed these cues during the execution of a reaching movement. Based on the cue, subjects had to either continue with the movement (go signal) or had to stop it (stop signal).

The results demonstrate that subjects could perform the correct motor action (go and stop trials) without being consciously aware of the content of the visual cue.

## Methods

Eleven healthy subjects (8 women, 3 men, aged 29±5 years (mean ± STD) without known neurological disorders and with normal or corrected to normal vision participated in the study. One subject, an experienced table tennis player, responded correctly (i.e. always above chance level, motor responses as well as certain answers in the verbalization protocol, see below) even for the visual cues with the lowest display duration. It was, in other words, not possible with the hardware at hand to present visual cues of sufficiently short duration to compromise conscious perception in this subject. We therefore excluded her from further analysis. All remaining subjects were right handed according to the Oldfield handedness inventory [12] and gave written informed consent to the experimental protocol. The study was performed according to the Declaration of Helsinki (1964) and approved by the local ethics committee of the University of Copenhagen and Freiburg (HA-2008-029).

### Experimental procedure

**Figure 1:**
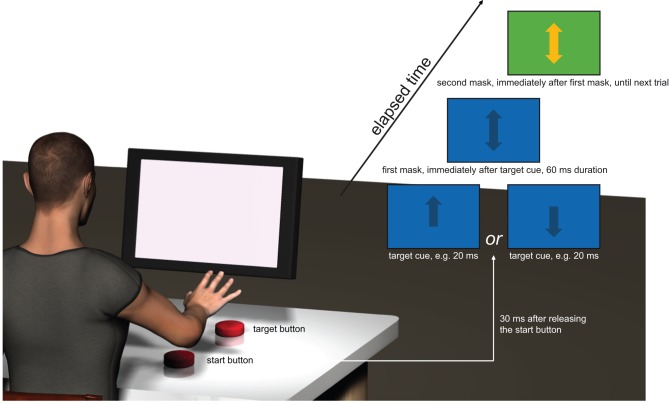
Sketch of the experimental setup. A visual cue, specifically an arrow pointing upwards or downwards, was presented after subjects released a start button (delay between releasing and presentation on the screen: 30 ms). The upward arrow indicated that subjects should continue to the target button, the downward arrow indicated that subjects should abort the movement. The visual cue, determining the response, was presented with different display durations, ranging from 20 ms to 300 ms. Two masks, presented immediately (delay below 1 ms) after the visual cue, degraded the perceptibility of the visual cue. The display duration for the target cue in this sketch was 20 ms. Note that, for all other display durations, the presentation of the masks was delayed (e.g. with a display duration of 40 ms for the target cue, the second mask appeared after 70 ms with respect to the release of the start button). Only in the *Verbalization protocol*, a tone was presented with a delay of 1 second after providing the target cue. Subjects had to verbalize what they saw before this tone.

The subjects were seated 60 cm in front of a computer screen (17 inch, Dell® E170S). A start button (10 cm length×10 cm width) was placed slightly to the right in front of the subjects. The right hand of the subjects, resting on this start button, had to be lifted and moved forward to a target button. The target button (10 cm length×10 cm width) was placed 50 cm in front of the start button. Releasing the start button produced a TTL-pulse and triggered a custom-built software programme (LabView based, National Instruments®, Austin, Texas), which was used to present the visual cues. 30 ms after the trigger, a 3 cm length×1 cm width target cue (upward or downward arrow) was shown on the computer screen. The colour of the target cue was dark blue, and the background colour was light blue. When the arrow pointed upwards, the subjects were instructed to reach and press the target button (termed go-condition), and when the arrow was pointing downwards, subjects had to abort the reaching movement (termed stop-condition). The target cue was followed and masked by two double arrows (an arrow pointing upwards and downwards), which had the same size as the target cue. The first double arrow appeared immediately (delay below 1 ms) after the target cue for 60 ms and showed the same colour (and background colour) as the target cue, the second one appeared directly afterwards and was visible until the next target cue appeared on the screen. The colour of the second mask was orange with a dark green background screen (see Fig. 1). In a preliminary study, we tested the combination of different cue, mask, and background colours on conscious perception. We found that, at a fixed display duration, this combination of colours served best to degrade conscious perception of the target cue. From this point, the variable we finally varied was the display duration of the target cue. The subjects were instructed to perform the reaching movements (which consisted of: releasing the button, perceiving the cue on the way to the target button, pressing or not pressing the target button, moving back to the initial start button) in a self-paced frequency. This frequency ranged between 0.2 and 0.4 Hz. When starting a reaching movement, the subjects were instructed to look straight ahead and fixate their gaze at the middle of the screen (i.e. the location where the target cue appeared). The TTL-pulse, elicited when releasing the start button, and a second TTL-pulse, elicited when subjects hit the target button, were recorded with custom-built software (LabView based, National Instruments®, Austin, Texas) and stored for off-line analysis. The second TTL-pulse served to analyse whether subjects made a go- or a stop-movement.

### Experimental protocols

Two different protocols were performed. In the first protocol, we tested the ability of the subjects to make correct motor responses to visual cues with different display durations (*Motor reaction*). In the second protocol, we tested the ability of the subjects to correctly verbalize the meaning of the visual cues with different display durations (*Verbalization*). The *Verbalization protocol* aimed to test at which display duration the subjects were unable to report conscious perception of the visual cue. We argued that if subjects were able to perform the *Motor reaction protocol* correctly in the absence of conscious perception (tested in the *Verbalization protocol*), this would indicate the capability of the subjects to have correct action selection based on subconscious visual information. Subjects started with the *Motor reaction protocol* or *Verbalization protocol* in a pseudorandomized order. Before executing these protocols, subjects accustomed to the task they had to execute.

### Customization

This protocol aimed to customize the subjects to the task. Therefore, the target cue was displayed for 300 ms while subjects performed reaching movements. This display duration was sufficiently long to enable conscious perception. 300 trials were performed, with brakes for 2 min after every 60 trials. The visual cue was randomized between ‘go’ and ‘stop’ condition. An important purpose of the customization was to make subjects accustomed to a movement time (time to complete a reaching movement), which was kept the same across the following trials and conditions. In a preliminary experiment, we found that the subjects kept the movement time as well as the timing between successive reaching movements similar once they were accustomed to the reaching task (this took typically 200 – 250 trials).

### Motor reaction protocol

In the motor reaction protocol, the subjects were instructed to perform reaching movements. They were told: “The only thing, which changes with respect to the customization period for the next 310 trials, will be a decrement in the display duration of the target cue in some of the trials”. Furthermore, they were told: “Make an immediate decision whether to go or to stop after you see the cue. Do what you see, and do not ponder about this decision.” There was a pause of 2 min after every block of 62 reaching movements. The display duration for go and stop trials were as follows: 20 ms, 40 ms, 60 ms, 80 ms, 100 ms, 120 ms, 160 ms. We ensured that the duration of the different cues was constant across trials and conditions in an additional experiment where we measured and analysed the durations of the cues on the screen using photodiodes (BPW 34, Siemens®, München, Germany; sample frequency: 2000 Hz). 10 trials were recorded for each of the display durations, both for go and stop cues (this makes 20 trials for each of the display durations). Furthermore, there were 160 reaching movements (go and stop) where the target cue was visible for 300 ms. Finally, there were 10 trials with no target cue but just the two masks (no cue condition). The different conditions were presented randomly but were equally distributed with respect to the number of upward arrows and downward arrows. Additionally, all conditions were equally distributed in each of the executed blocks (consisting of 62 trials).

### Verbalization protocol

In a second protocol, it was tested when, i.e. at which display duration, the subjects had conscious perception of the target cues presented to them. Therefore, they were instructed to place their hand on the start button and release it like in the *Motor reaction protocol*. However, instead of a motor response after releasing the button (go or stop movement) they were told to verbalize the direction of the target cue. When the subjects perceived the arrow clearly they reported “up” or “down” for arrow upwards and downwards. When they were not sure about whether they clearly perceived the direction, they said “guess up” or “guess down”. The self-paced frequency of the reaching movements in this protocol was kept the same like in the *Motor reaction protocol*. Importantly, subjects had to answer within one second before receiving a tone (500 Hz, 100 ms) after the target cue appeared. If the answer had come too late, the trial would have been disregarded. This was actually never the case in the present experiment, meaning that the time to respond was sufficient for all trials in all subjects. The following display durations were tested in this protocol: 20 ms, 40 ms, 60 ms, 80 ms, 100 ms, 120 ms, and 160 ms, respectively. Each of the display durations was tested 20 times (10 times arrow up, and 10 times arrow pointing downwards).

### Data analysis and statistics

The number of correct responses was calculated for all conditions (different display durations, including separation of go and stop trials in the *Motor reaction protocol*). In the *Verbalization protocol*, the number of correct answers was assigned to what subjects were guessing and what they were certain about.

A Two-way repeated-measures ANOVA was used to analyse whether the number of correct responses (go and stop) were different in the *Motor reaction* versus the *Verbalization protocol* using the within-subject factors protocol (*Motor reaction* versus *Verbalization*) and display duration (20 ms, 40 ms, 60 ms, 80 ms, 100 ms, 120 ms, and 160 ms). Post-hoc (Bonferroni) corrected paired-student T-tests were used to indicate differences between the two protocols for each of the display durations.

An additional repeated-measures ANOVA with the within-subject factors task (go/stop) and display duration (20 ms, 40 ms, 60 ms, 80 ms, 100 ms, 120 ms, and 160 ms) served to test whether subjects performed better in the go-trials or stop-trials in the *Motor reaction protocol*.

In a further analysis of the *Verbalization protocol*, the individual display duration was determined at which the subjects decided wrong in at least 50% of the trials. 50% equals the probability of pure guessing. All correct verbalized responses of this protocol (the ones where subjects were guessing and the ones where they were certain about) were taken into account. For instance, subject A named 100% of the arrows correct at the display duration of 160 ms, 90% at 120 ms, 80% at 100 ms, 60% at 80 ms, and 50% at 60 ms. In subject A, 60 ms was the display duration of interest, i.e. this subject needed more than 60 ms to consciously perceive the stimulus. In a second step, we calculated the number of correct responses at the display duration of interest in the *Verbalization protocol* and also the number of correct responses at the same display duration in the *Motor reaction protocol*. Finally, the group values of correct responses in the *Verbalization protocol* were compared with the group values of correct responses in the *Motor reaction protocol* by paired Student's T-tests.

Data are reported as group mean values ± standard deviation (S.D.). SPSS 19.0 (SPSS® Inc., USA) was used for the statistical analysis.

## Results

### Motor reaction versus Verbalization

**Figure 2:**
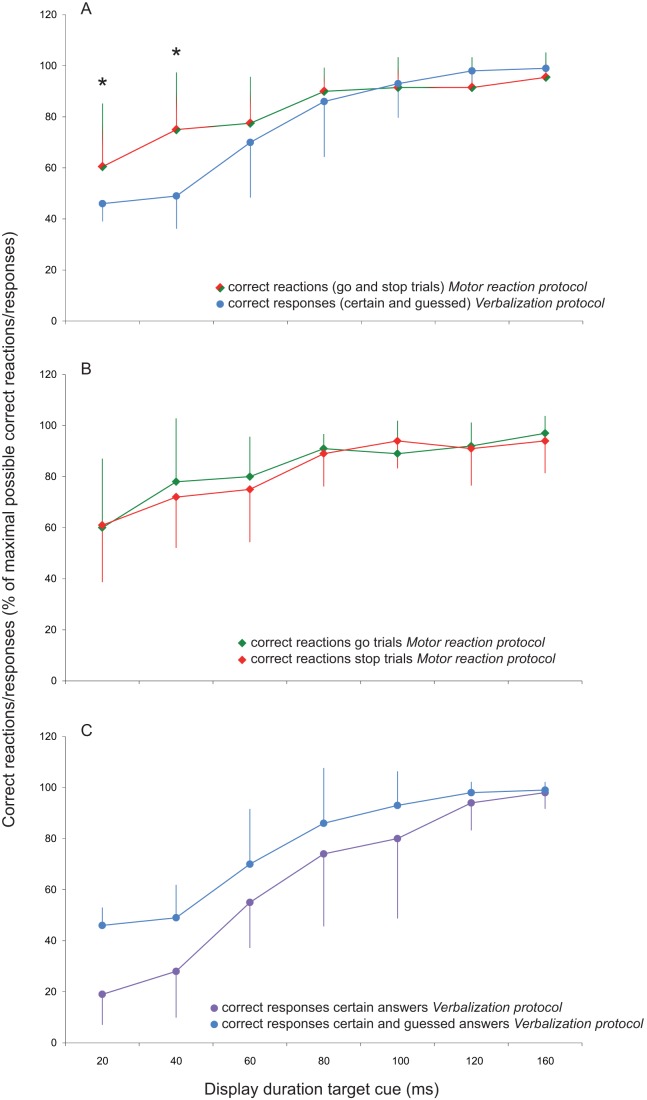
Group mean values of the results obtained in the *Motor reaction protocol* and the *Verbalization protocol* for each of the tested display durations. Bars indicate S.D. **A.** Shown are the correct reactions in the *Motor reaction protocol* versus the correct responses in the *Verbalization protocol* as a percentage of the maximal possible correct reactions/responses. **B.** *Motor reaction protocol*. Correct reactions obtained for go versus stop trials. Note that the number of correct reactions was not different between go and stop trials at each of the display durations. **C.** *Verbalization protocol*. Correct responses obtained for certain answers versus certain answers plus guesses. Consequently, differences between the certain answers and the certain answers plus guessed answers at each of the display durations correspond to portion of guessed answers. Note that the number of guessed answers increased with shorter display durations.

The repeated-measures ANOVA, testing whether subjects responded differently between the two protocols, revealed no significant effect for the factor ‘protocol’ (F_1,9_  = 3,19; p = 0.11) but a significant effect for the factor ‘display duration’ (F_6,54_  = 44,98, p<0.001) and a significant ‘protocol’ × ‘display duration’ interaction (F_6,54_  = 7.55, p<0.001, Fig. 2). This means that the number of correct responses were significantly different between different display durations in both protocols. Furthermore, indicated by the ‘protocol’ × ‘display duration’ interaction, there was a significant difference of the correct responses between the two protocols for specific display durations. Post-hoc pairwise comparisons were performed to reveal these display durations. Significant differences in the number of correct responses between the two protocols were seen only at two display durations, namely at a display duration of 20 ms (p<0.01), and 40 ms (p<0.01), respectively. At these two display durations, subjects achieved more correct responses in the *Motor reaction protocol* compared to the *Verbalization protocol* (certain plus guessed answers) (see Fig. 2).

In an additional analysis, the individual display duration where the subjects made correct responses in 50% or less of the trials was determined and the number of correct responses during reaching (*Motor reaction protocol*) at this display duration was compared with that during *Verbalization* (see Methods for details). The level of 50% was chosen because it equals the distribution of correct and incorrect responses by pure guessing. The corresponding display duration for this level was 44±17 ms across subjects (grand mean value ± STD). When taking the individually determined display duration of correct answers in the verbalization trials (43±8% correct answers) and comparing this value with the correct responses in the *Motor reaction protocol*, a significantly better performance was evident for the latter (73±24% correct trials, p<0.01).

### Was it easier to go than to stop?

The number of correct responses for the go-trials and the stop-trials in the *Motor reaction protocol* is shown in Fig. 2. A repeated measures ANOVA with the within-subject factors task (go/stop) and display duration (20 ms, 40 ms, 60 ms, 80 ms, 100 ms, 120 ms, and 160 ms) revealed a significant effect for display duration (F_6,54_  = 23.13, p<0.001) but not for task (F_1,9_  = 0.07, p = 0.79), and no significant display duration × task interaction (F_6,54_  = 0.45, p = 0.84). This means that the correctness of the response was not dependent on the specific reaction (go versus stop).

The average time for the subjects from receiving the visual cue to press the target button in the go trials was 821 ± 205 ms.

### Presenting no cue resulted in a 50% chance to perform the go

As mentioned before, there was an additional test condition in the *Motor reaction protocol* where no cue was presented. In this condition, only the mask was shown on the screen. None of the subjects did notice that there was no cue presented to them. Based on the equal distribution of go and stop signals in the task there should be a 50% chance for the subjects to press the target button, provided that reaching and stopping possess similarly strong movement representations. This was actually the case as the target was hit in 53±17% of the trials (Student's T-test testing against 50%: p = 0.64).

## Discussion

The main finding of the present study was that subjects achieved more correct responses in the *Motor reaction protocol* than in the *Verbalization protocol* at two distinct display durations of the visual cues guiding the responses. These display durations were 20 ms, and 40 ms, respectively. The *Verbalization protocol* aimed to test for conscious perception of the visual cues. Our findings indicate that, at these two short display durations, subjects were significantly more likely to make a correct motor response than to consciously perceive and verbalize the meaning of the visual cue. Regarding the relationship between display duration and conscious perception, Fig. 2 indicates that at long cue durations subjects were more certain of which cue was displayed, whereas they were uncertain of their perception and had to guess at short visual cue durations. Furthermore, in an additional, more individualized, analysis, we calculated at which display duration the individual subject responded wrong in at least 50% of the trials in the *Verbalization protocol*. As 50% of right and wrong answers equal the probability of pure guessing, it can be proposed that the subject was unable to consciously perceive the cue at this display duration. However, when the same subject received visual cues at the identical display duration during the reaching movement (*Motor reaction protocol*), his or her probability to respond correctly to the cue was significantly enhanced for both go and stop trials. Taken all of this together, we argue that subjects in the present study were able to choose the correct motor action in response to a subconscious visual cue.

Our findings support and extend the observations made in previous studies [11], [13]. For instance, in the study by Christensen et al. [13], subjects made choice reactions while their primary visual cortex was excited using transcranial magnetic stimulation. Transcranial magnetic stimulation degraded conscious perception of visual information. Despite this degradation, subjects made correct motor responses. The reason for this phenomenon was argued to be a disruption of the neural pathways causing conscious visual perception. In contrast, it was hypothesized that pathways processing subconscious visual information were left intact and triggered the reaction. There are, however, important differences between our study and the study of Christensen et al. [13]. In the latter experiment, subjects had to choose between two alternative motor responses whereas they had to choose between an action versus no action in the present study. A second, even more important difference between the current study and the study of Christensen et al. [13] is that the neural processing was artificially affected through transcranial magnetic stimulation in the latter one. A problem, with respect to the interpretation of the results, when interfering with the processing of visual information lies in the interconnectivity of the brain. More specifically, there is no conclusive argument that transcranial magnetic stimulation, although targeting the primary visual area, is not affecting areas responsible for factors modulating consciousness per se. One of these factors is attention, which has been shown to correlate with more specific activation of the cerebral cortex [14]. Here, transcranial magnetic stimulation could be disruptive to the neural processes underlying attention. As a consequence, the visual stimulus may have been processed similarly as without stimulation, but attention could have been affected. In contrast, the effect we saw was solely based on the perceptibility of the visual stimulus.

In addition to the artificial disruption of the neural processing like in the study of Christensen et al. [13], there exists another approach to investigate whether healthy humans can react to subconscious visual information. This approach is based on the modulation of the perceptibility of the visual information. One of the most frequently used forms of this approach is backward masking or priming [15], [16], [17]. In backward masking, a strong visual cue is presented with the consequence that a preceding weaker visual stimulus (in terms of contrast and/or display duration) cannot be consciously separated from the strong stimulus. If the preceding stimulus matched the target cue (consistent prime), the response was shown to be quicker in simple reaction time tasks. The opposite, i.e. a prolongation of the reaction time, was shown when the prime cue did not match the target cue (inconsistent prime) [1]. The experimental design in the study of Taylor and McCloskey [11] was based on this technique and is described in detail in the introduction. In contrast to the present study, Taylor and McCloskey [11] used a mask, which was not neutral. This means that both the subconscious and the conscious stimuli comprised the same information content. When the subconscious, weak stimulus appeared on the left side, the strong conscious stimulus also appeared on this side and the subjects had to flex their left elbow. When the subconscious and conscious stimuli appeared on the right side, subjects made an elbow flexion on the right. Thus, subjects had theoretically always the possibility to select the correct response based on the strong stimulus. It might therefore be assumed that the time delay between the subconscious (weak LED flash) and the conscious (strong LED flash) visual stimulus was not sufficient to detect the first weak stimulus as a separate response but it was likely ample to facilitate the response. Thus, it might be possible that a subconscious stimulus alone would have been too weak to initiate a reaction. Therefore, this study leaves the question open whether a subconscious stimulus on its own is sufficient to trigger appropriate motor responses in a choice reaction task. In order to tackle this question, we tested whether subconscious visual information alone is sufficient to correctly choose the appropriate motor response. Although masking was also applied in the present study, the mask (strong stimulus) could not trigger or influence any response, as the crucial information content for choosing the correct response was not displayed by this stimulus. In other words, we used a neutral mask, which appeared always at the same spot. The information to continue (arrow up) or to stop the movement (arrow down) was exclusively hidden in the preceding weak (subconscious) stimulus, which appeared always at the same location on the screen, too.

It has been proposed repeatedly, that voluntary complex arm movements in the primate and in human need to be, at least partly, controlled by neocortical networks [18], [19], also involving processing of subconscious information [20], [21]. Consequently, the subconscious visual cue triggering the motor reaction in the present study might be processed in high, neocortical, areas. Some studies hypothesized that the human brain has the capability to process subconscious and conscious visual information by two different “streams” [22], [23], whereas other, recently published articles, argue against this hypothesis [24], [25]. Studies promoting this hypothesis differentiate between a ventral stream responsible for fast reactions. The dorsal stream, passing from the primary visual cortex to the posterior parietal lobe involving the primary visual area of the neocortex, was ascribed to conscious perception and decision making [22]. Time constraints may allow usage of the ventral stream. With lower threshold detection, the ventral stream may bypass time-consuming conscious perception and trigger fast movements. However, it has to be mentioned that it is still under debate whether or not there exist two different streams for processing different aspects of visual perception. Therefore, conclusions about the neural processing of unconscious information in the present study cannot be drawn.

A potential confounding factor of the present study is that the level of conscious perception may have changed between the executions of the two different protocols. The differences in conscious perception might therefore be responsible for the fact that subjects could perform correct motor reactions to the visual cues but were significantly less able to correctly verbalize the scene. Although many factors modulate conscious perception, it seems nevertheless unlikely that they were responsible for the observed findings. One of these modulating factors is certainly attention. Potentially, the subjects may have become tired over time and/or did not pay attention to the screen in some of the trials. Hence the conscious perception of the visual cue would be different. However, based on the study design we argue that differences in the attention between the protocols were unlikely. First, the subjects were explicitly instructed throughout the experiment to focus on the screen when the visual cue appeared. Further and more important, the subjects triggered the visual cue by themselves when releasing the start button. Consequently, they were asked to start a reaching trial only when paying attention to the screen. This was true for the *Motor reaction protocol* as well as for the *Verbalization protocol*. The decision to let the subjects trigger the visual cue actively in the *Verbalization protocol* (meaning that the cue appeared when the subjects decided to release the start button and that it was not presented at a fixed time interval, e.g. every 4 seconds) was primarily based on our consideration of attention. With e.g. a fixed interval of 4 seconds, the chance that subjects would miss a visual cue based on reduced attention would have been higher than with an active triggering where subjects automatically focused on the task at the time they started the trial. In addition to the active triggering of the visual cue, the subject had long breaks in-between trials to minimize the effect of fatigue and therefore prevent different levels of attention throughout the experiment. Finally, the order of the two protocols was pseudorandomized.

Another factor modulating consciousness is memory extinction. When receiving information, only parts of it may be memorized for a longer period of time. Some of it extinguishes and/or converts (referring to the content) over time. Consequently, it might be argued that the extinction of the memory of the visual cue may be different between the *Motor reaction protocol* and the *Verbalization protocol* because the time between receiving the visual cue and the reaction to it may have been different. In order to counteract this problem, we used an auditory cue in the *Verbalization protocol*. The subjects had to answer within 1 second and only trials within this time frame were counted. We argue that this delay was sufficiently short to prevent significant memory extinction, which could account for the observed differences of the results between the *Motor reaction protocol* and the *Verbalization protocol*.

When establishing the threshold for the duration of the visual cue, we focused on the discrimination threshold since this was functionally relevant for the motor task. It may be that the detection threshold (i.e. the ability to recognize the presence of a stimulus without discriminating between the two different cues) was even lower, but this was not tested in the present study. This would mean that, at short cue durations, the presence of a visual stimulus would still be perceived despite impaired discriminability.

A last issue worth to consider is that there was no possibility to avoid motor responses when testing conscious perception. The verbal response in the Verbalization protocol is thus also a motor reaction, although engaging different muscles than those involved in the behavioural response. It is therefore possible that subjects in the present study did consciously perceive the visual information but did not report it because this information did not pass from areas processing speech to their motor system.

### Conclusion

In the present study, subjects made correct motor reactions based on subconscious visual information. This finding strengthens the findings made by Taylor and McCloskey [11]. However, in contrast to previous studies, where the information content of subconscious and conscious (mask) stimuli were the same, the mask in our study was neutral and therefore reactions were solely dependent on the information content of the subconscious target cues. This is of great importance as previous studies could not clarify whether motor actions are indeed selected and performed based on the subconscious cue or whether the subconscious cue only facilitated the response of the subsequent stronger stimulus (mask). Thus, the present study highlights that motor choice reactions are possible solely in response to subconscious cues.
